# How to interpret Methylation Sensitive Amplified Polymorphism (MSAP) profiles?

**DOI:** 10.1186/1471-2156-15-2

**Published:** 2014-01-06

**Authors:** Jaroslav Fulneček, Aleš Kovařík

**Affiliations:** 1 Institute of Biophysics, Academy of Sciences of the Czech Republic, v.v.i., Královopolská 135, Brno CZ-612 65, Czech Republic

**Keywords:** MSAP, DNA methylation, Methylcytosine, *Msp*I, *Hpa*II, Hemimethylated site, Data interpretation

## Abstract

**Background:**

DNA methylation plays a key role in development, contributes to genome stability, and may also respond to external factors supporting adaptation and evolution. To connect different types of stimuli with particular biological processes, identifying genome regions with altered 5-methylcytosine distribution at a genome-wide scale is important. Many researchers are using the simple, reliable, and relatively inexpensive Methylation Sensitive Amplified Polymorphism (MSAP) method that is particularly useful in studies of epigenetic variation. However, electrophoretic patterns produced by the method are rather difficult to interpret, particularly when *Msp*I and *Hpa*II isoschizomers are used because these enzymes are methylation-sensitive, and any C within the CCGG recognition motif can be methylated in plant DNA.

**Results:**

Here, we evaluate MSAP patterns with respect to current knowledge of the enzyme activities and the level and distribution of 5-methylcytosine in plant and vertebrate genomes. We discuss potential caveats related to complex MSAP patterns and provide clues regarding how to interpret them. We further show that addition of combined *Hpa*II + *Msp*I digestion would assist in the interpretation of the most controversial MSAP pattern represented by the signal in the *Hpa*II but not in the *Msp*I profile.

**Conclusions:**

We recommend modification of the MSAP protocol that definitely discerns between putative hemimethylated mCCGG and internal CmCGG sites. We believe that our view and the simple improvement will assist in correct MSAP data interpretation.

## Background

The DNA of most eukaryotic organisms contains 5-methylcytosine (mC) residues, which represent important epigenetic information involved in the regulation of gene expression during various developmental processes such as cell differentiation, imprinting, or X chromosome inactivation [1-3]. DNA methylation also contributes to genome stability by repressing harmful genetic elements [4]. The DNA methylation pattern may be changed by diet or stressful external conditions [5,6]. In plants, epialleles occurring naturally or after genetically/chemically induced epimutations may produce heritable phenotypic diversity supporting adaptation and evolution [7].

In a genome, only a fraction of cytosine residues is postreplicatively methylated by DNA (cytosine-5) methyltransferases [8]. The level of mC varies among individual species, and mC distribution along DNA is not uniform. Within an individual, the tissues or cell types differ in mC distribution. Repetitive sequences usually contain more mC than genic sequences [9]. Methylation of promoters leads to transcriptional inactivation of linked genes in most cases; the function of evolutionary conserved gene body methylation is still unknown [10,11]. Vertebrate DNA is predominantly methylated in CG dinucleotides [12-14]; however, in plant DNA, a cytosine in any sequence context can be methylated [15] with decreased frequency for CG, CHG, and CHH motifs (H = A or T or C; [16]).

To identify changes in the methylation of genomic DNA connected with biological processes or different types of treatments, a convenient method for mC detection should be used. Recently, several genome-wide methods were developed to analyze mC [17] based on bisulfite modification [16] or immunoprecipitation [18]. Because these methods are entirely dependent on detailed knowledge of the genome sequence, many scientists use Methylation Sensitive Amplified Polymorphism (MSAP), particularly in non-model organisms. The MSAP method based on digestion with methylation-sensitive restriction endonucleases followed by amplification of restriction fragments is independent on the availability of genome sequence information and has been used frequently to analyze DNA methylation changes in plants and animals [19-21]. The MSAP procedure was first introduced by Reyna-Lopez and co-workers [22]. In their modification of the original amplified fragment length polymorphism protocol [23], frequent cutter *Mse*I was replaced by methylation-sensitive *Msp*I and *Hpa*II restriction enzymes. However, the choice of these particular enzymes may lead to ambiguous interpretation of MSAP data, and some findings may be inconsistent with our current knowledge of mammalian and plant DNA methylation. Here, we reviewed 16 years of experience with the MSAP technique and attempted to explain the ambiguous characteristics of some restriction patterns considering the methylation sensitivities of restriction enzymes and known methylation frequencies of various sequence motifs in vertebrate and plant genomes. We present a modification of the MSAP protocol that would help to interpret the patterns of the most commonly used enzymes, *Msp*I and *Hpa*II.

## Results and discussion

### Principle of MSAP

In the original MSAP protocol [22], genomic DNA is divided into two parts, and each part is digested with *Eco*RI, which recognizes the GAATTC target site and is thought to be negligibly influenced by DNA cytosine methylation, and one of the methylation-sensitive *Msp*I or *Hpa*II isoschizomers, which can both cleave CCGG sequences. *Msp*I can cleave non-methylated CCGG sequences and hemi (mC in one DNA strand only) or fully methylated CmCGG sequences but not hemi and fully methylated mCCGG and mCmCGG sequences [22,24]. *Hpa*II is presumed to digest only non-methylated CCGG sequences and hemimethylated mCCGG sequences from all possible methylated CCGG variants [22,25]. The DNA samples digested with *Eco*RI and *Msp*I or *Eco*RI and *Hpa*II are ligated to two dsDNA adapters compatible with *Eco*RI- and *Msp*I/*Hpa*II-generated ends. Ligated fragments are preamplified using non-selective or pre-selective primers complementary to the adapters followed by amplification with a pair of selective primers (these are one- to three-base extended variants of non-selective or pre-selective primers at 3′ ends). Such amplification produces a reduced population of fragments that are separated in denaturing polyacrylamide gels. Visualization of fragments is usually accomplished by the introduction of a radioactively or fluorescently-labeled primer during a selective amplification step. In the gel, each DNA sample is represented by two neighboring lanes of fragments resulting from *Eco*RI, *Msp*I- and *Eco*RI, *Hpa*II-digested DNA (M and H lane, respectively). Thus, four MH fragment pattern variants "+ +, - -, + -, - +", referring to the presence (+) or absence (-) of a fragment, can be observed for each position in the gel. The (+, +) pattern (a fragment of definite length visualized in both the M and H lanes) is attributed to digestion by both enzymes at a non-methylated CCGG site. The (-, -) pattern indicates inhibition of digestion with both enzymes at a fully methylated mCmCGG site when another treated sample shows the presence of a fragment at that position. The (-, -) pattern may also represent a mutated site when genetically distinct samples are compared. The (+, -) pattern representing a fragment of definite length visualized in the M but not in the H lane corresponds to digestion with *Msp*I but not *Hpa*II and refers to the presence of a CmCGG site. Finally, the (-, +) pattern seems to be quite difficult to interpret, particularly in plant genomes where methylation occurs in both the CCG and CG motifs.

### Methylation sensitivity of restriction enzymes

The interpretation of MSAP data is predominantly based on known restriction enzyme (RE) activities at recognition sequences modified by methylation. Data concerning the methylation sensitivity of REs and corresponding literature can be found at the website of The Restriction Enzyme Database (REBASE; http://rebase.neb.com; [26]). At REBASE, 18 different references were identified concerning *Msp*I and/or *Hpa*II enzyme activities at the recognition sequence containing 5-mC residues (13 for *Msp*I and 14 for *Hpa*II; http://rebase.neb.com).

The CCGG recognition site can theoretically occur in 10 differentially methylated forms (Table 1), and not all of them were tested in *Msp*I/*Hpa*II activity assays. In addition, different methylated forms may provide identical *Msp*I and *Hpa*II digestion profiles, suggesting only limited ability of the MSAP method to detect all methylation changes at CCGG sites. As follows from the function of bacterial *Hpa*II and *Msp*I restriction-modification systems, CCGG sites should be resistant to digestion when the inner cytosines (for *Hpa*II) or outer cytosines (for *Msp*I) are methylated by cognate methyltransferases [27,28]. Simultaneously, postreplicatively generated sites methylated only at one DNA strand (termed hemimethylated) should also be resistant to digestion. It is known that *Msp*I can digest *Hpa*II-methylated sites (CmCGG) [29,30]; however, *Hpa*II cannot digest *Msp*I-methylated sites (mCCGG) [24,31]. Inconsistency has been found regarding the sensitivity of *Hpa*II to methylation of the outer C (mCCGG) in the hemimethylated state. While earlier work reported cleavage of hemimethylated mCCGG sites with *Hpa*II [25], other studies did not completely support such activity [24,31-33]. This discrepancy may be explained by the known slow *Hpa*II nicking activity of the non-methylated DNA strand [24] possibly combining with impurity-driven nonspecific nuclease activity in some samples. Such DNA degradation might be supported by the observed lower intensity of *Hpa*II digestion products compared with non-digested or *Hpa*II-methylated DNA templates [25]. Indeed, Korch and Hagblom found smearing of *Hpa*II-digested products after longer incubation or a higher enzyme concentration and suggested contaminating unspecific nuclease(s) in all *Hpa*II enzyme preparations they used [32]. The current view supports *Hpa*II nicking activity of the non-methylated but not the methylated strand in hemimethylated mCCGG sequences [31]. However, there is continuing controversy that the specificity of the *Hpa*II enzyme and its ability to digest hemimethylated mCCGG sequences may be dependent on the enzyme concentration and incubation time.

**Table 1:** Frequency, effect on cleavage and expected MSAP profile of ten different methylated forms of CCGG site

**Site**	**C C G G**	^**3**^**C**^**m**^**C G G**	**C**^**m**^**C G G**	**C**^**m**^**C G G**	**C**^**m**^**C G G**	^**m**^**C C G G**	^**m**^**C C G G**	^**m**^**C C G G**	^**m**^**C**^**m**^**C G G**	^**m**^**C**^**m**^**C G G**
	**G G C C**	**G G C C**	**G G**^**m**^**C C**	**G G**^**m**^**C**^**m**^**C**	**G G C**^**m**^**C**	** G G C C**	** G G C**^**m**^**C**	** G G**^**m**^**C**^**m**^**C**	**G G C C**	**G G**^**m**^**C**^**m**^**C**
^1^Frequency	Low	Low	High	High	^9^Xlow	Xlow	Xlow	Xlow	Xlow	High
*Msp*I	^4^+	+	+	-	-	-,n	-	-	-,n	-
*Hpa*II	+	^6^-,^8^n	-	-	-	-,^8^N	-	-	-,n	-
^2^MH pattern	^5^(+,+)	(+, -)	(+, -)	^7^(-, -)	(-, -)	(-, -)	(-, -)	(-, -)	(-, -)	(-, -)

^1^Frequency of theoretical methylated forms is estimated for plant genomes [16,37].

^2^MSAP profile pattern of a fragment with the site after digestion with *Msp*I (M) and *Hpa*II (H).

^3^(^m^C) represents 5-methylcytosine residue.

^4^(+) sequence is cut.

^5^(+) a band is present.

^6^(-) sequence is not cut.

^7^(-) a band is absent.

^8^n and N denote negligible and slow nicking of non-methylated strand, respectively.

^9^Xlow denotes extremely low.

Although the *Eco*RI enzyme recognizing GAATTC is used as an enzyme that is not sensitive to methylation in MSAP, *Eco*RI did not digest GAATTmC sequences when cytosines at both strands are methylated [34,35]. Thus, *Eco*RI digestion may be inhibited by overlapping C methylation, indicating that not all changes in MH patterns always reflect changes in the methylation status of *Msp*I/*Hpa*II sites.

### Frequency of mC at CCGG sites in plant and vertebrate genomes

Despite earlier reports regarding the high frequency of mCCG methylation in plant genomes, [36] the more recent data support the following frequencies: CmCGG > mCmCGG> > mCCGG [16,37] which is in accord with DNA methylation and demethylation studies in plants [38,39]. Although the expected frequency of *Msp*I/*Hpa*II sites is one per 256 bp, the exact spacing is highly variable depending mostly on the GC content of a particular chromosomal region. In regions with closely spaced CCGG sequences, the supposed internal CmCGG site(s) of the *Hpa*II-*Eco*RI fragment would be digested by *Msp*I, and the resulting shorter *Msp*I-*Eco*RI fragment may be lost during selective amplification or may be too short for electrophoretic detection. This finding is consistent with the observation that most (-, +) MH type fragments are long, while most (+, -) MH type fragments are typically short [40]. If so, all isolated *Hpa*II fragments of the (-, +) MH type should contain at least one additional internal CCGG site; indeed, this was reported for all such sequenced fragments [41]. Internal CCGG sequences were also identified in *Hpa*II-specific fragments obtained from restriction landmark genome scanning of *A. thaliana* and rice, and their CmCGG methylation status was confirmed by Southern blot hybridization and/or by polymerase chain reaction (PCR) [42,43]. More evidence for the presence of internal CmCGG site(s) in the fragments of the (-, +) MH type comes from MSAP analysis of seven swine tissues where approximately 26.2% and 25.7% of MH patterns were of the (+, -) and (-, +) type, respectively [21]. Considering that vertebrate genomes are methylated almost exclusively at CG sites and a nearly equal number of (+, -) and (-, +) MH profiles, we may expect an identical origin of these two patterns; essentially, the occurrence of CmCGG sequences only. This presumption is certainly valid also for the other two vertebrate species analyzed by MSAP [44,45].

### Hyper- and hypomethylation may not be easily deciphered from MSAP patterns

**Figure 1:**
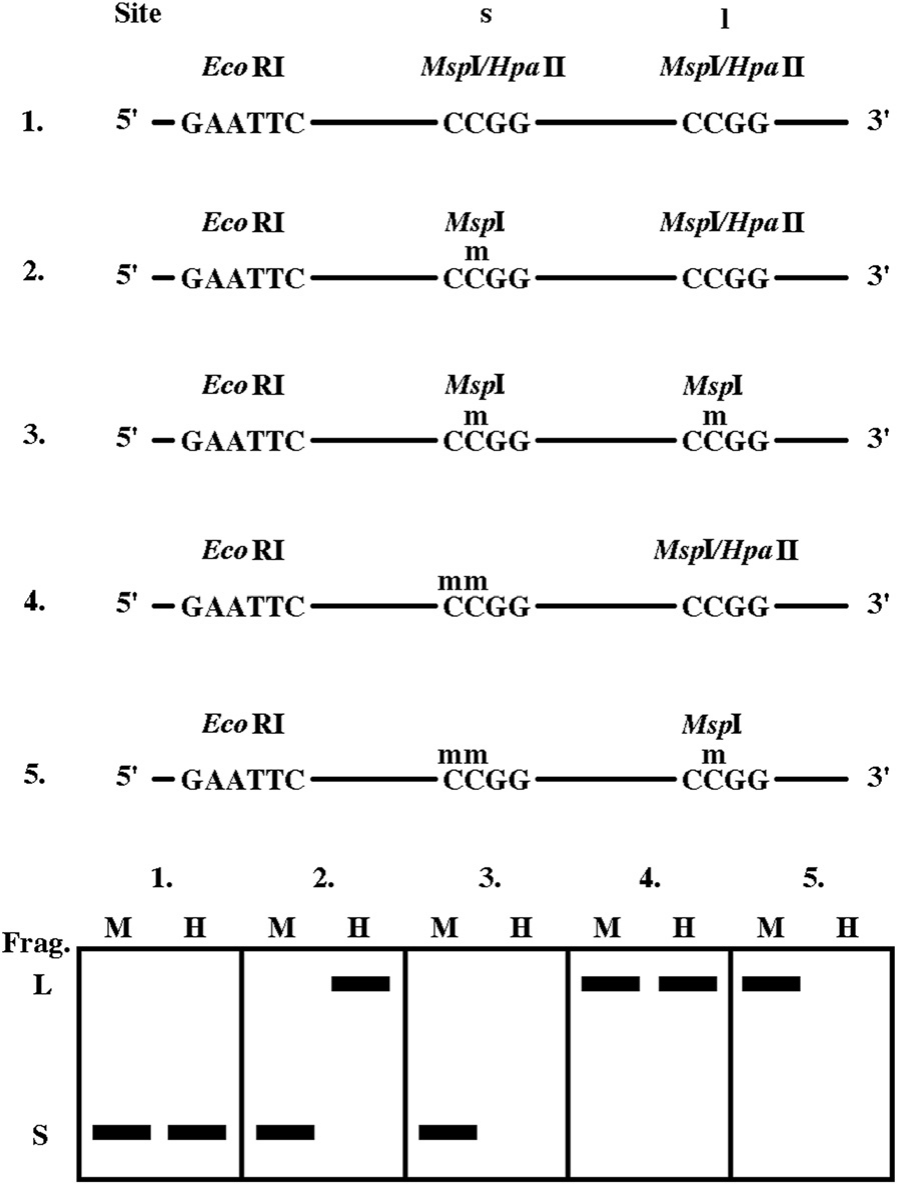
**DNA with two closely spaced** ***Msp*I/*Hpa*II sites in five methylation states and the expected MSAP signal.** A model DNA molecule with two closely spaced CCGG sites is depicted in five different methylated variants. To simplify, only one DNA strand is shown, and a symmetrical methylation pattern is expected. Variants 1–3 are found in both vertebrate and plant DNA, whereas variants 4 and 5 are plant specific. The methylation state of the two CCGG sites (Site s and l) influences digestion by *Msp*I and *Hpa*II. The expected MSAP MH patterns of the generated short (S) and long (L) fragments (Frag.) are drawn in boxes for the five methylated variants. Note that digested S and L DNA fragments may be amplified by different selective primers, and, if so, they are not visible together on one MSAP profile.

Particularly for sequences with closely spaced *Msp*I/*Hpa*II sites, up to half of the different MSAP pattern changes are ambiguous and potentially misinterpreted either at the level of methylation change or as a change in nucleotide sequence (Table 2). Considering a model DNA molecule with closely spaced CCGG sites occurring in five different methylation variants (Figure 1), variants 1–3 are found in both vertebrate and plant DNA while variants 4 and 5 are plant specific. First, we would expect amplification of the long fragment (L) due to the specific combination of preselective and selective primers. Variant 2 will show a (-, +) MH pattern (due to an internal CmCGG site) that will be changed either by hypomethylation (variant 1) or hypermethylation (variant 3) into a (-, -) MH pattern. A shift from the (-, +) to the (+, +) MH pattern is not expected in vertebrate DNA samples; however, it may be quite frequent in plant samples (variant 4) and is caused by methylation of the outer cytosine in the internal CmCGG site. However, the most commonly used interpretation of the shift from the (-, +) to (+, +) MH pattern is hypomethylation of a putative mCCGG site.

**Table 2:** **Possible changes in MSAP MH profiles from Figure**1**and their interpretation**

**Plants**			
^1^MH1 → MH2	methylation change	MSAP pattern (Figure 1)	^7^interpretation
^2^(-, -) → (+, +)	^3^s(mCmC → CC)	^4^S^5^(4 → 1)	ambiguous
	^6^s(mCmC → CC) and l(CmC → CC)	S(5 → 1)	
	s(CC → mCmC)	L(1 → 4)	
	s(CmC → mCmC) and l(CmC → CC)	L(3 → 4)	
(-, -) → (+, -)	s(mCmC → CmC)	S(4 → 2) or S(5 → 3)	ambiguous
	s(mCmC → CmC) and l(CC → CmC)	S(4 → 3)	
	s(mCmC → CmC) and l(CmC → CC)	S(5 → 2)	
	s(CmC → mCmC)	L(3 → 5)	
	s(CC → mCmC) and l(CC → CmC)	L(1 → 5)	
(-, -) → (-, +)	l(CmC → CC)	L(3 → 2)	ambiguous
	s(CC → CmC)	L(1 → 2)	
(+, +) → (-, -)	s(CC → mCmC)	S(1 → 4)	ambiguous
	s(CC → mCmC) and l(CC → CmC)	S(1 → 5)	
	s(mCmC → CC)	L(4 → 1)	
	s(mCmC → CmC) and l(CC → CmC)	L(4 → 3)	
(+, +) → (+, -)	s(CC → CmC)	S(1 → 2)	methylation
	s(CC → CmC) and l(CC → CmC)	S(1 → 3)	
	l(CC → CmC)	L(4 → 5)	
(+, +) → (-, +)	s(mCmC → CmC)	L(4 → 2)	hypomethylation
(+, -) → (-, -)	s(CmC → mCmC)	S(2 → 4) or S(3 → 5)	ambiguous
	s(CmC → mCmC) and l(CC → CmC)	S(2 → 5)	
	s(CmC → mCmC) and l(CmC → CC)	S(3 → 4)	
	s(mCmC → CmC)	L(5 → 3)	
	s(mCmC → CC) and l(CmC → CC)	L(5 → 1)	
(+, -) → (+, +)	s(CmC → CC)	S(2 → 1)	hypomethylation
	s(CmC → CC) and l(CmC → CC)	S(3 → 1)	
	l(CmC → CC)	L(5 → 4)	
(+, -) → (-, +)	s(mCmC → CmC) and l(CmC → CC)	L(5 → 2)	hypomethylation
(-, +) → (-, -)	s(CmC → CC)	L(2 → 1)	ambiguous
	l(CC → CmC)	L(2 → 3)	
(-, +) → (+, +)	s(CmC → mCmC)	L(2 → 4)	methylation
(-, +) → (+, -)	s(CmC → mCmC) and l(CC → CmC)	L(2 → 5)	methylation
**Vertebrates**			
MH1 → MH2	sequence change	MSAP pattern (Figure 1)	interpretation
(-, -) → (+, +)	^8^new CCGG	-	mutation
(-, -) → (+, -)	new CmCGG	-	mut. + met.
(-, -) → (-, +)	l(CmC → CC)	L(3 → 2)	ambiguous
	s(CC → CmC)	L(1 → 2)	
(+, +) → (-, -)	CCGG loss	-	mutation
(+, +) → (+, -)	s(CC → CmC)	S(1 → 2)	methylation
(+, +) → (-, +)	new internal CmCGG	-	mut. + met.
(+, -) → (-, -)	CmCGG loss	-	mutation
	new internal CmCGG	-	
(+, -) → (+, +)	s(CmC → CC)	S(2 → 1)	hypomethylation
	s(CmC → CC) and l(CmC → CC)	S(3 → 1)	
(+, -) → (-, +)	(CmC → CC) and new int. CmCGG	-	mutation
(-, +) → (-, -)	s(CmC → CC)	L(2 → 1)	ambiguous
	l(CC → CmC)	L(2 → 3)	
(-, +) → (+, +)	internal CmCGG loss	-	mutation
(-, +) → (+, -)	(CC → CmC) and int. CmCGG loss	-	mutation

^1^MH1 and MH2 represent the original (control) and a changed MSAP profile, respectively.

^2^- and + in parentheses denote the absence and the presence of a band in *Msp*I (M) and *Hpa*II (H) lanes.

^3^s and l indicate restriction sites in Figure 1 (the "methylation change" and "sequence change" columns).

^4^S and L indicate specific MSAP fragments (the "MSAP pattern column") according their lengths from Figure 1.

^5^The numbers in parenthesis correspond to the methylation states of the sequence and the MSAP profiles in Figure 1.

^6^Some MSAP profile changes are explained by two methylation changes (both s and l *Msp*I/*Hpa*II sites).

^7^Interpretation for each MH1→MH2 profile change is noted in the interpretation column.

^8^Majority of possible MSAP profile changes in vertebrates are explained by gain or loss of the restriction site and therefore are expected to be very rare.

### A modified MSAP protocol improves problematic interpretation of the (-, +) MH pattern

**Figure 2:**
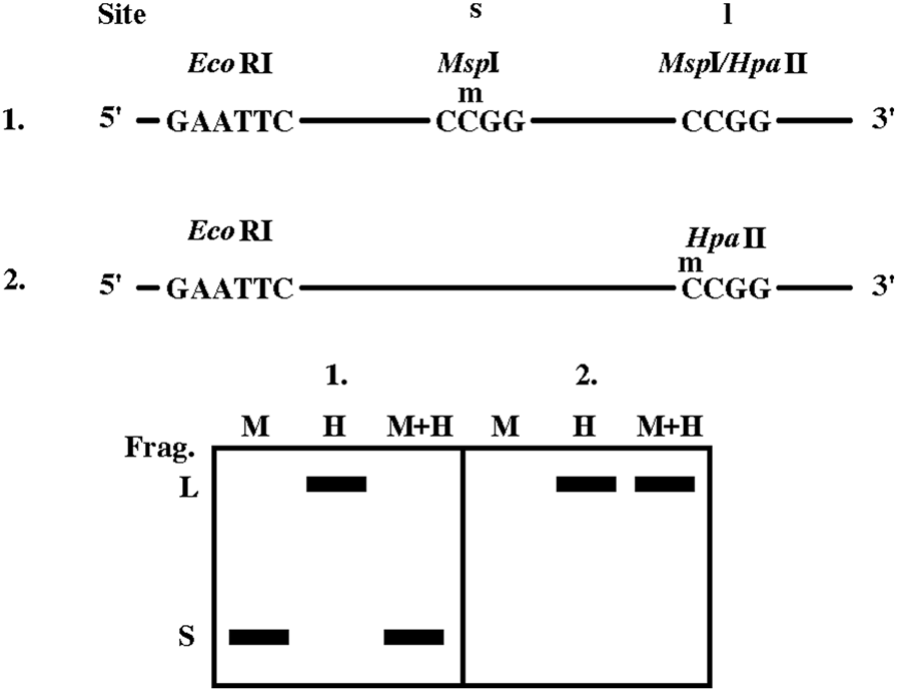
**Verification of the internal CmCGG site in (-, +) MH** ***Eco*RI-*Hpa*II fragments by the addition of the M + H profile.** Two different DNA molecules producing a (-, +) MH pattern for the long (L) fragment can contain either an internal CmCGG site (1) or hemimethylated mCCGG site (2). Combined digestion by both *Msp*I and *Hpa*II enzymes (M + H lanes) distinguish between the two molecules. Note that digested short (S) and long (L) DNA fragments may be amplified by different selective primers; if so, they are not visible together on one MSAP profile.

**Figure 3:**
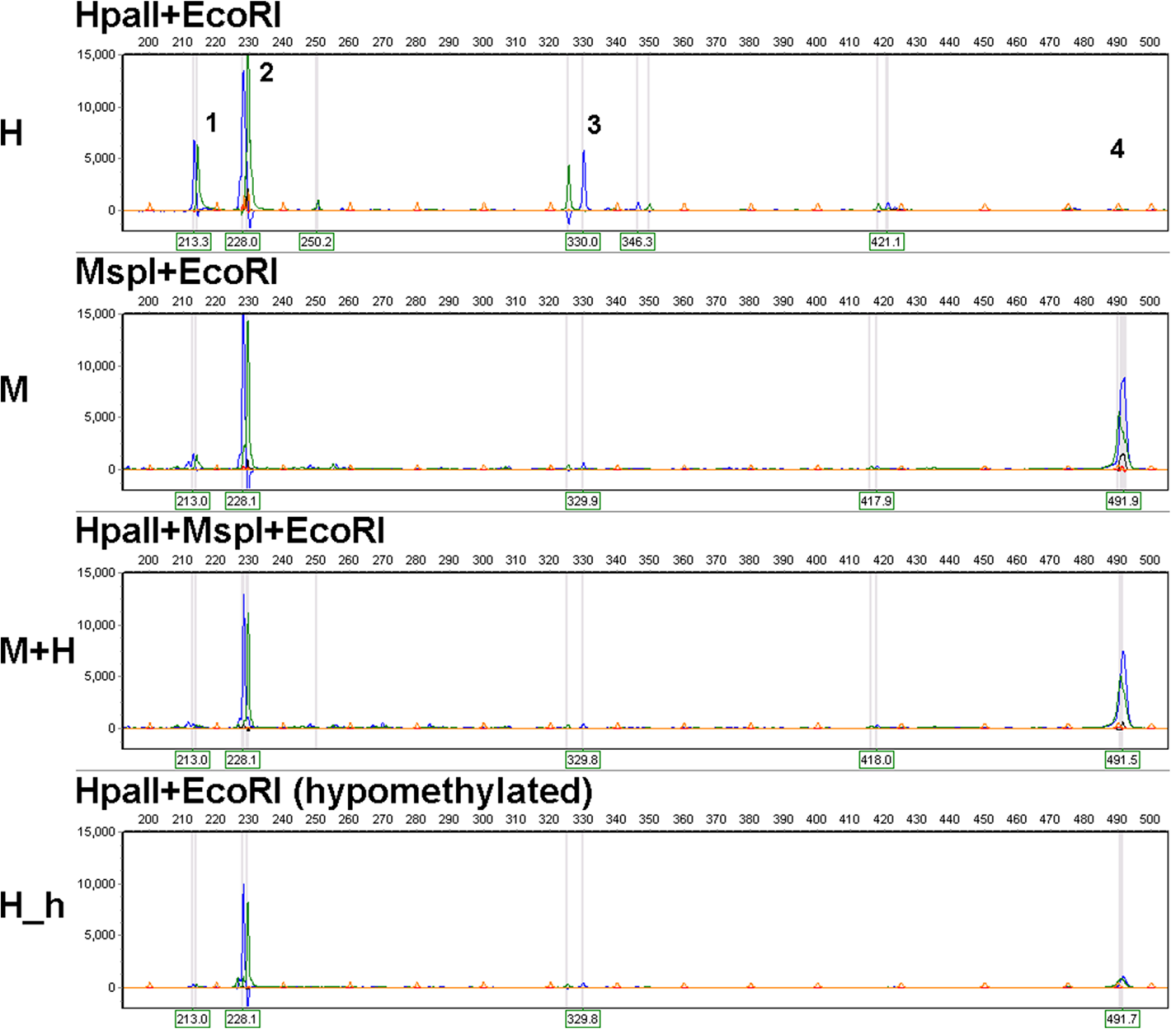
**Example of MSAP electropherograms documenting assessment of (-, +) MH patterns using** ***Msp*I +** ***Hpa*II double digestion.** Fragments 1 and 3 represent the (-, +) MH pattern, fragment 2 represents the (+, +) MH pattern, and fragment 4 represents the (+, -) MH pattern. Note that fragments 1 and 3 disappear after application of the combination of the *Hpa*II, *Msp*I and *EcoR*I enzymes (M + H panel) or after DNA hypomethylation (H_h panel) by 100 μM DHPA [48]. Each restriction fragment is represented by two peaks (green and blue) derived from two complementary DNA strands (differently labeled selective primers were used). The presence of both peaks at the same position or in close proximity (note that the mobility of complementary strands of a DNA fragment may not be equal; [23]) indicates that the fragment had both *Eco*RI and *Msp*I/*Hpa*II ends.

As stated above, in plant genomes, the (-, +) MH pattern seems to be particularly problematic because it may be interpreted as two different situations: (i) the cutting of the hemimethylated mCCGG sites with *Hpa*II but not *Msp*I and (ii) the presence of internal CmCGG site(s) between the cleaved distal CCGG and the *Eco*RI site (Figure 2). Correct interpretation of the (-, +) MH pattern is important for classification of methylation changes that may involve both hypermethylation and hypomethylation. Here we show that introduction of a "third" lane containing DNA digested with both *Msp*I and *Hpa*II enzymes (M + H lane) may be highly informative. The persistence of the "H only" band leading to the (-, +, +) (M, H, M + H, in this order) pattern would support hemimethylation of the mCCGG. By contrast, loss of the "H only" band (-, +, -) indicates the presence of an internal CmCGG site. We decided to test this theoretical construct by analysis of control and hypomethylated tobacco DNA (Figure 3, Additional file 1: Figure S1). A typical (-, +) MH pattern is shown in Figure 3 containing two *Hpa*II fragments (1, 3) without corresponding peaks in the *Msp*I lane. Neither fragment 1 nor fragment 3 was resistant to combined *Hpa*II + *Msp*I cleavage (-, +, -), indicating that fragments 1 and 3 contained two consecutive differentially methylated CCGG sites: one proximal to the *Eco*RI site methylated at CmCGG and one distal non-methylated CCGG site. The products of *Msp*I digestion are not visualized probably because they are too short and/or are not amplified by the selective primers. In hypomethylated DNA, we observed a loss of fragments 1 and 3 after *Hpa*II digestion (Figure 3, panel H_h), further supporting the presence of internal CmCGG site(s) in the fragments. The only truly non-methylated fragment is fragment 2 that is visualized in the H, M and H + M lanes and indicates proper DNA digestion.

Dinucleotide "mCG only" methylation is localized in coding regions known to possess high GC content and a relatively high frequency of CCGG sites compared with the rest of the plant genome. Thus, the (-, +, -) patterns are likely formed by fragments having their origin in coding regions. By contrast, the (-, +, +) patterns, indicating mCCG methylation, would originate prevalently from repetitive non-coding regions because trinucleotide mCHG methylation is primarily localized to satellite repeats and transposons [46]. Thus, the proposed MSAP modification utilizing combined M + H digestion may help to determine the genomic origin of methylated fragments. The mCmCGG sites, which are common in plant heterochromatin, will not be digested with either *Msp*I or *Hpa*II (-, -, - pattern). This may explain why heterochromatic sequences are usually underrepresented in eluted MSAP fragments.

Regarding vertebrate DNA, which is methylated almost exclusively in CG sequences, we infer that the (-, +) MH pattern may not be interpreted as mCCGG methylation [21] but rather as internal CmCGG sites.

Because there is no entirely methylation-insensitive isoschizomer cutting at the CCGG motif, discrimination between methylation (epigenetic change) and mutation (genetic change) may be difficult, particularly among individuals of different genotypes. Therefore, employing other restriction sites may be an interesting option. Among others, *Sau*3AI (sensitive to methylation at GATmC sites), *Eco*T22I (ATGmCAT, [47]), *Nla*III (mCATG), and *Scr*FI (CmCNGG) may be considered. For *Sau*3AI and *Nla*III, methylation-insensitive *Mbo*I and *Cvi*AII izoschizomers are available, respectively.

## Conclusions

Clearly, the MSAP method is a reliable, inexpensive, and relatively simple genome-wide method for the identification of genome regions with putative changes in DNA cytosine methylation in response to environmental and developmental stimuli. While the interpretation of the (+, -) and (+, +) *Msp*I/*Hpa*II patterns is straightforward, the presence of an *Hpa*II signal without a concomitant *Msp*I signal (-, +) is more ambiguous. In the latter case, we recommend modifying the MSAP protocol by including an additional lane in which the DNA sample is digested with a combination of both enzymes. Such improvement helps to distinguish between di- and trinucleotide methylation and between methylation in coding and noncoding areas of the plant genome.

## Methods

### DNA samples

We used DNA samples isolated from tobacco seedlings treated with 0, 10, and 100 μM 9-(*S*)-(2,3-dihydroxypropyl)-adenine (DHPA) and analyzed previously by *Msp*I/*Hpa*II digestion and Southern blot hybridization [48]. DHPA is a reversible competitive inhibitor of *S*-adenosylhomocysteine-hydrolase that elevates the intracellular SAH concentration [49]. SAH competes with *S*-adenosylmethionine (SAM), leading to inhibition of most SAM-dependent methyltransferases. In plants, DHPA treatment induces hypomethylation of CHG sites preferentially (CHG DNA methylation represents a metabolic bottleneck due to its dependence on histone methylation) and also hypomethylation of CG sites to some extent at elevated DHPA concentrations [48].

### MSAP procedure

The MSAP procedure was performed in two technical replicates starting from the same DNA sample. DNA (1 μg) was digested in NEB buffer 1 with 10 U of *Hpa*II (NEB) in a 20-μl reaction volume for 3 hours at 37°C. Ten microliters of the reaction mixture was transferred to 10 μl of NEB 2 buffer with 5 U of *Msp*I (NEB) and incubated for 3 hours at 37°C. Simultaneously, 0.5 μg of the same original DNA sample was incubated in NEB 2 buffer with 5 U of *Msp*I (NEB) in a 10-μl reaction volume for 3 hours at 37°C. Next, 10 μl of *Eco*RI buffer (NEB) with 5 U of *Eco*RI (NEB) were added to 10-μl reactions (*Hpa*II and *Msp*I digestions) and 5 μl of 2.5× *Eco*RI buffer (NEB) with 5 U of *Eco*RI (NEB) were added to a 20-μl *Hpa*II + *Msp*I reaction volume followed by incubation for an additional 1.5 hours at 37°C. *Eco*RI_ADAPTER1(F) 5′-CTCGTAGACTGCGTACC-3′ (10 μM)/ *Eco*RI_ADAPTER2(R) 5′-AATTGGTACGCAGTCTAC-3′ (10 μM) and *Hpa*II/*Msp*I_ADAPTER1(F) 5′-GATCATGAGTCCTGCT-3′ (100 μM)/ *Hpa*II/*Msp*I_ADAPTER2(R) 5′-CGAGCAGGACTCATGA-3′ (100 μM) were used to prepare 5 μM of *Eco*RI and 50 μM of *Msp*I/*Hpa*II adapters, respectively, by mixing appropriate oligonucleotides and incubating at 98°C for 5 min followed by slow cooling in a polystyrene box (for approximately 6 hours). Six microliters of digested DNA samples was added to 3.75 μl of mix comprising 1 μl of 5 μM *Eco*RI adapter (5 pmol), 1 μl of 50 μM *Msp*I/*Hpa*II adapter (50 pmol), 1 μl of fresh 10× T4 DNA ligase buffer, and 0.75 μl of sterile redistilled water. After mixing and heating to 37°C, 0.25 μl of T4 DNA ligase (100 U, NEB) was added, and the mix was incubated at 37°C for 3 hours followed by incubation at 20°C overnight. Non-ligated adapters were removed, and ligated DNA was purified using the NucleoSpin Gel and PCR Clean-up Kit (Macherey-Nagel). DNAs were eluted in 2 × 10 μl of 5 mM Tris-Cl (pH 8), and 3 μl was used in 10-μl preselective amplification reactions with *Eco*RI_A 5′-GACTGCGTACCAATTCA-3′ and *Hpa*II/*Msp*I_T 5′-ATGAGTCCTGCTCGGT-3′ primers at 200 nM concentration each. In addition, the preselective reactions contain DynazymeII buffer, 125 μM dNTPs, and 0.5 U of DynazymeII DNA Polymerase (Thermo Scientific). The conditions of the preselective amplification were as follows: 72°C for 5 min, 94°C for 3 min followed by 30 cycles of 94°C for 30 s, 56°C for 30 s, and 72°C for 2 min. A final step at 60°C for 10 min was also added. Five microliters of preselective products was checked by electrophoresis in 2% agarose gels (visible as a smear from 100 to 1000 bp) and the remaining 5 μl was diluted by adding 50 μl of redistilled water. The diluted preselective product (2.5 μl) was amplified using fluorescently labeled *Eco*RI_ACT[HEX] (green peaks in Figure 3 and Additional file 1: Figure S1) and labeled *Hpa*II/*Msp*I_TTC[6-FAM] (blue peaks in Figure 3) or *Hpa*II/*Msp*I_TAG (Additional file 1: Figure S1) selective primers (200 nM each) in a 12.5-μl total reaction volume containing KAPA buffer B, 125 μM dNTPs, and 0.5 U of KAPA Taq DNA Polymerase (KAPA Biosystems). The conditions of the selective amplification were as follows: 94°C for 3 min, 13 cycles of 94°C for 30 s, 65°C for 30 s reduced by 0.7°C per cycle, and 72°C for 2 min followed by 24 cycles of 94°C for 30 s, 56°C for 30 s, and 72°C for 2 min. A final step at 72°C for 10 min was also added. The product of selective amplification (5 μl) was checked by 2% agarose gel electrophoresis. Next, 1.5 μl of the selective amplification product was added to a mix of 10 μl of deionized formamide for capillary electrophoresis (Genomac) and 0.3 μl of GMC GT500-L DNA standard (Genomac) that is fluorescently labeled with LIZ dye (orange peaks in Figure 3 and Additional file 1: Figure S1). DNA was denaturated by heating at 98°C for 3 min followed by cooling on ice. Denatured DNA was separated using the fragment analysis option in the ABI Prism 3100 Genetic Analyzer. The obtained electropherograms were aligned according the signal of the DNA standard, analyzed and visualized using GeneMarker version 1.80 (SoftGenetics).

## Authors’ contributions

JF and AK designed the study, JF performed the experiments. JF and AK analyzed and interpreted the data as well as drafted the manuscript. Both authors read and approved the final manuscript.

## Supplementary Material

Additional file 1: Figure S1 MSAP analysis of DNA samples isolated from tobacco seedlings treated with 0 μM (DHPA 0), 10 μM (DHPA 10) and 100 μM (DHPA 100) 9-(*S*)-(2,3-dihydroxypropyl)-adenine (DHPA). Click here for file
