# Supplementary material for: How to interpret Methylation Sensitive Amplified Polymorphism (MSAP) profiles?
Source: BMC Genet. 2014 Jan 6;15:2. doi: 10.1186/1471-2156-15-2 (PMC3890580; doi:10.1186/1471-2156-15-2)
Supplement: Additional file 1: Figure S1 — MSAP analysis of DNA samples isolated from tobacco seedlings treated with 0 μM (DHPA 0), 10 μM (DHPA 10) and 100 μM (DHPA 100) 9-(S)-(2,3-dihydroxypropyl)-adenine (DHPA). [file 1471-2156-15-2-S1.pdf]

How to interpret Methylation Sensitive Amplified Polymorphism (MSAP) Profiles?

Jaroslav Fulneček, Aleš Kovařík

Additional file 1: Figure S1

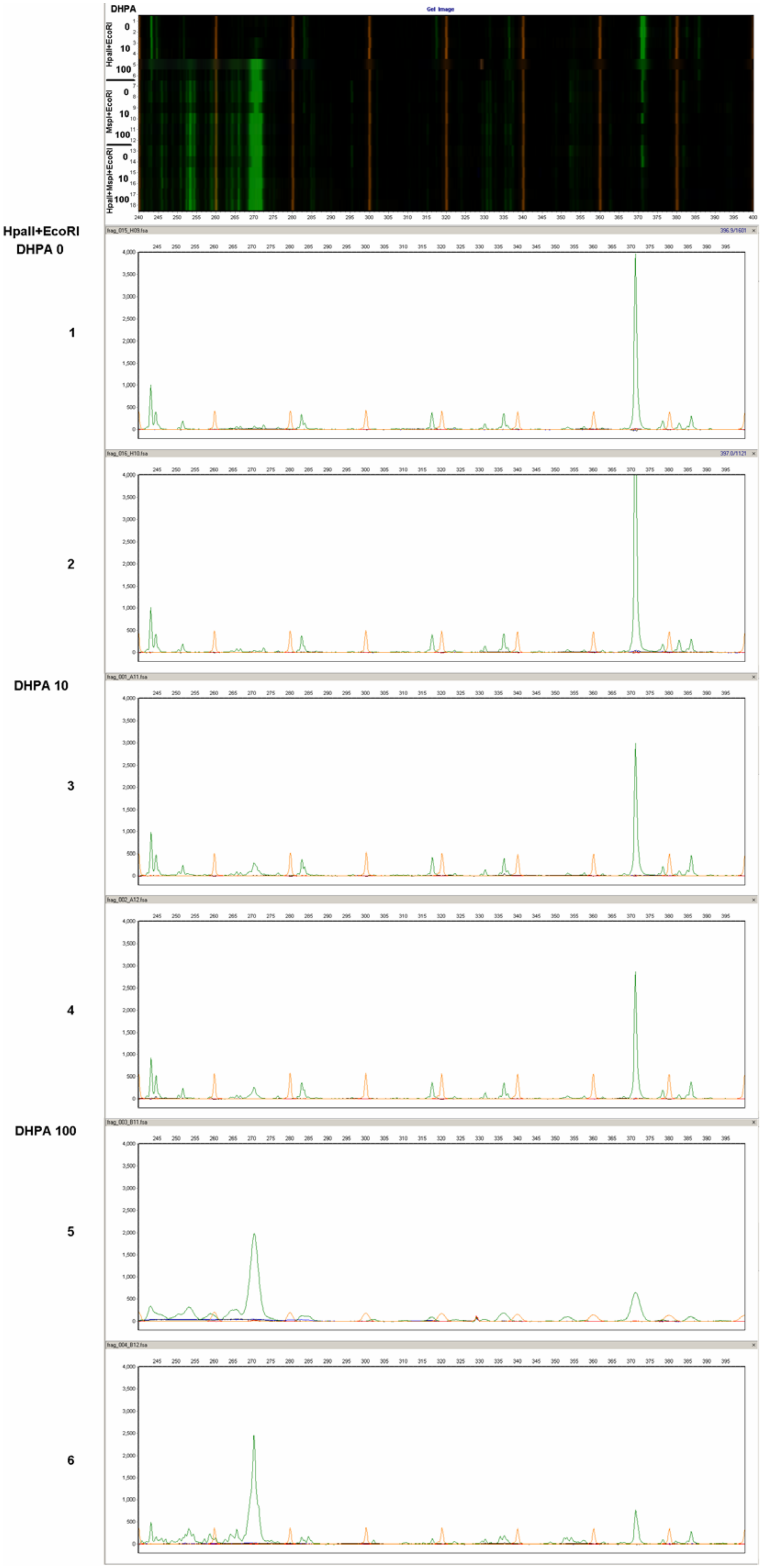

DHPA
Gel Image
HpaII+EcoRI
MspI+EcoRI
HpaII+MspI+EcoRI
0
10
100
HpaII+EcoRI
DHPA 0
1
2
DHPA 10
3
4
DHPA 100
5
6

MspI+EcoRI
DHPA 0

7

8

DHPA 10

9

10

DHPA 100

11

12

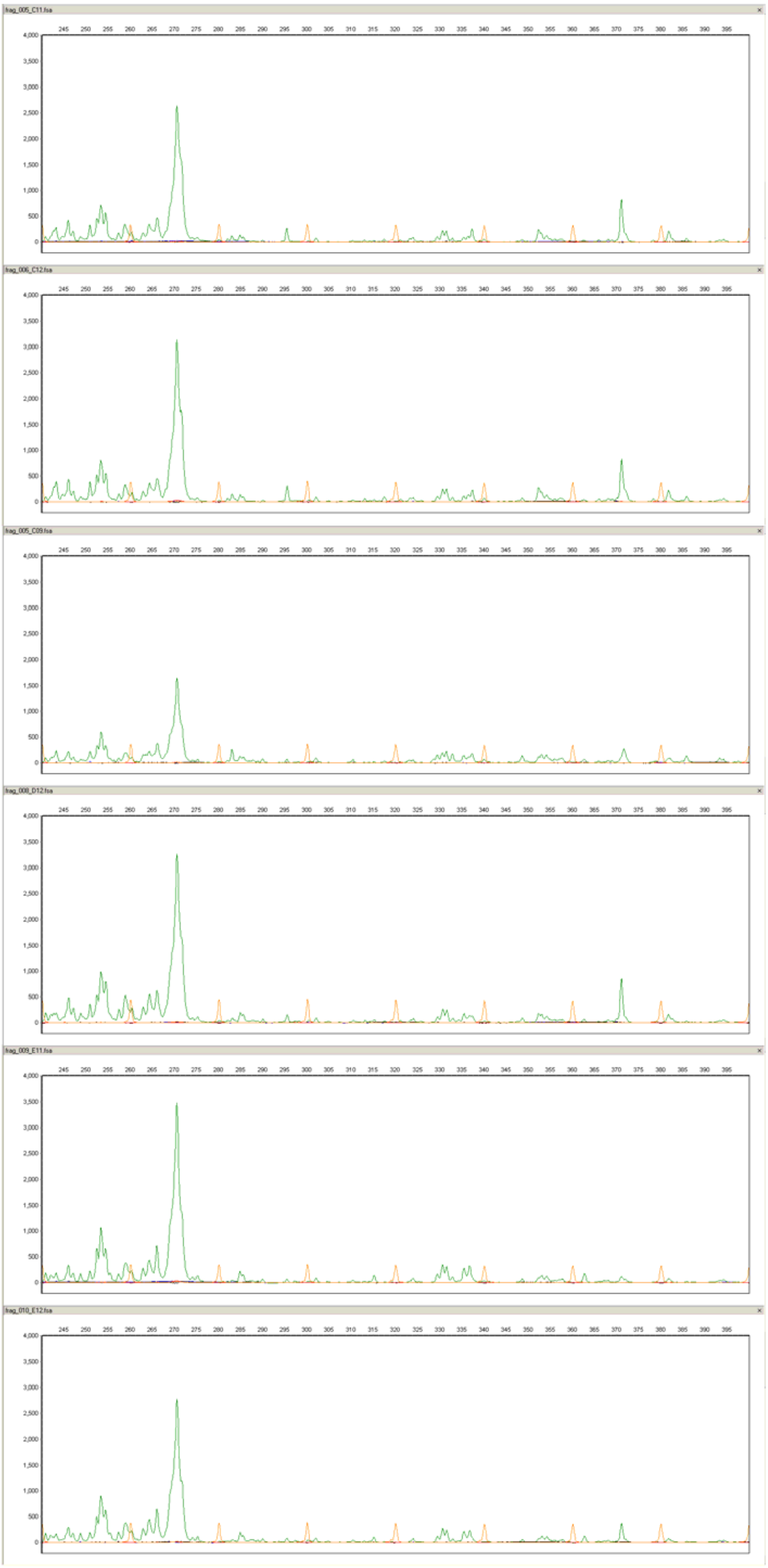

HpaII+MspI +EcoRI

DHPA 0

13

14

DHPA 10

15

16

DHPA 100

17

18

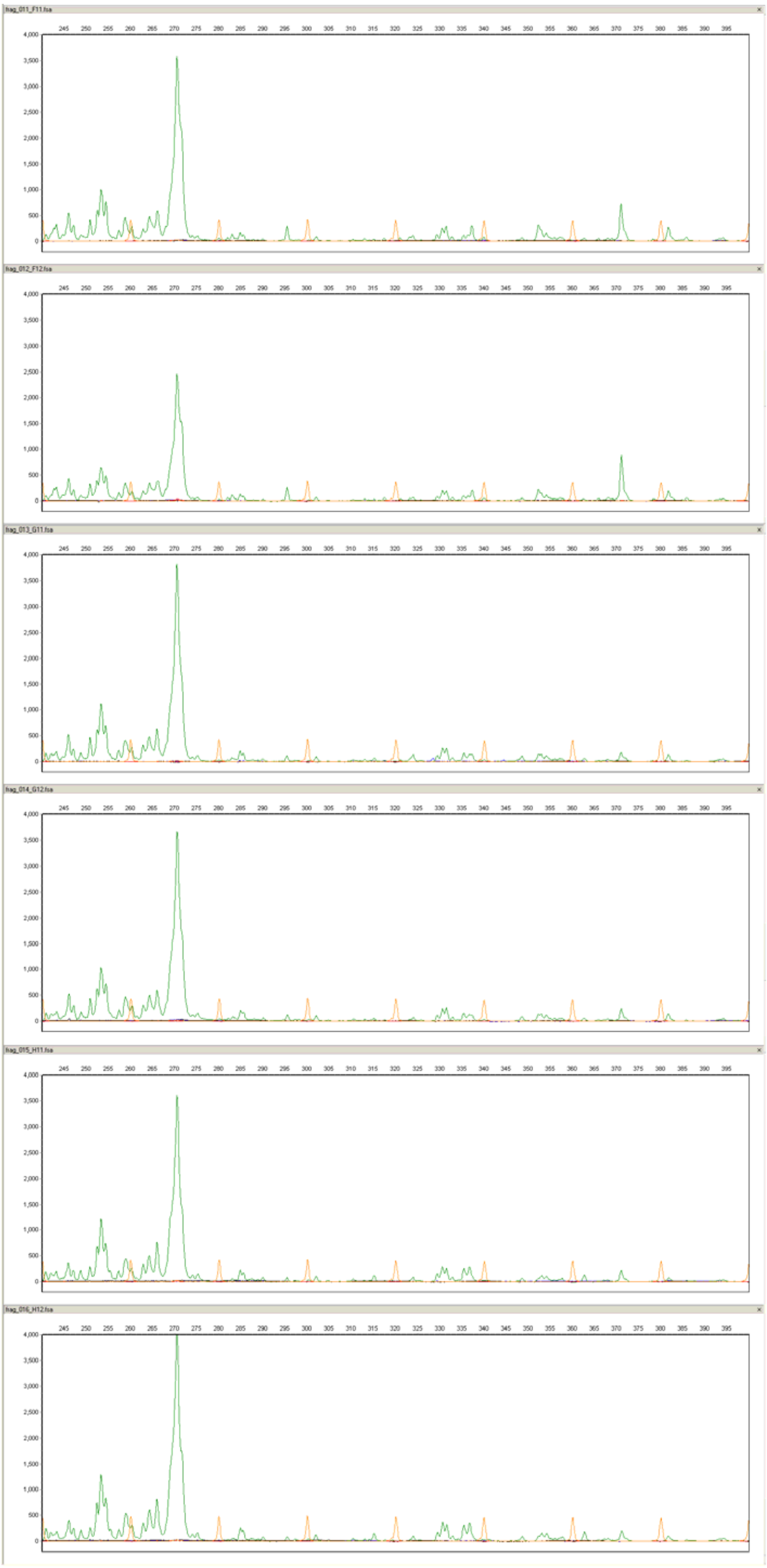

Figure S1 - MSAP analysis of DNA samples isolated from tobacco seedlings treated with 0 µM (DHPA 0), 10 µM (DHPA 10) and 100 µM (DHPA 100) 9-(*S*)-(2,3-dihydroxypropyl)-adenine (DHPA; [1]).

DHPA preferentially induces hypomethylation of CHG sequences and also some CG sequences at elevated concentrations (100 µM). *Hpa*II+*Eco*RI, *Msp*I+*Eco*RI and *Hpa*II+*Msp*I+*Eco*RI combinations of enzymes were used to identify the *Hpa*II_*Eco*RI fragments with internal CmCGG site(s). *Eco*RI_A, *Hpa*II/*Msp*I_T and *Eco*RI_ACT[HEX], *Hpa*II/*Msp*I_TAG pairs of primers were used in pre-selective and selective amplifications, respectively. GMC GT500-L DNA standard (Genomac; orange peaks) was added to fluorescently labeled products of selective amplification (green peaks) and after denaturation, ss DNA fragments were separated using ABI Prism 3100 Genetic Analyzer. Obtained data were aligned and visualized using GeneMarker version 1.80 (SoftGenetics LLC). Black panel represents gel image of 18 samples. MSAP procedure was performed in two technical replicates starting from identical DNA sample. Comparable profiles of technical replicates suggest sufficient reproducibility of applied procedure. 371 nt peak observed after *Hpa*II+*Eco*RI digestion is very high representing probably multi-copy sequence (panels 1, 2). It is significantly lower in DNA sample isolated from seedlings treated with 100 µM DHPA (panels 5, 6) but not in DNA sample isolated from seedlings treated with 10 µM DHPA (panels 3, 4). It is also significantly lower in all DNA samples digested with both *Msp*I+*Eco*RI (panels 7-12) and *Hpa*II+*Msp*I+*Eco*RI (panels 13-18) combinations of enzymes. These results indicate that 371 nt peak represents most probably a fragments with internal CmCGG site(s) which were either digested by *Hpa*II after hypomethylation or digested by *Msp*I. This may be also applicable for 243 nt peak. A small fraction of fragments corresponding to the 371nt peak had probably internal mCmCGG site as suggested by comparing *Msp*I digestions of non-treated and DHPA-treated samples. Peaks 270 nt and 253 nt represent fragments with CmCGG site at the ends. Due to three selective bases, the probability that 371 nt and 270 nt peaks represent fragments of the same origin is roughly 1.56 %. Note identical profiles of samples digested with *Msp*I+*Eco*RI and *Hpa*II+*Msp*I+*Eco*RI combinations of enzymes which indicate that *Hpa*II did not digest more sites than *Msp*I in this case at least.

References

1. Fulnecek J, Matyasek R, Votruba I, Holy A, Krizova K, Kovarik A: **Inhibition of SAH-hydrolase activity during seed germination leads to deregulation of flowering genes and altered flower morphology in tobacco.** *Mol Genet Genomics* 2011, **285**:225-236.
